# Synthesis of 1-isopropyl-3-acyl-5-methyl-benzimidazolone Derivatives and Their Antimicrobial Activity

**DOI:** 10.3390/ijms14046790

**Published:** 2013-03-26

**Authors:** Nan Xu, Chunnan Yang, Xinqi Gan, Shaopeng Wei, Zhiqin Ji

**Affiliations:** College of Plant Protection, Northwest A&F University, Yangling 712100, Shaanxi, China; E-Mails: xunan@nwsuaf.edu.cn (N.X.); yangchunnan@nwsuaf.edu.cn (C.Y.); ganxinqi@nwsuaf.edu.cn (X.G.); weishaopeng@nwsuaf.edu.cn (S.W.)

**Keywords:** 5-methyl**-**benzimidazolone, *N*-acylated derivatives, antimicrobial activity

## Abstract

A series of *N*-acylated analogues of 1-isopropyl-3-acyl-5-methyl-benzimidazolone were synthesized. Bioassay results indicated that analogues **5-07** and **5-19** exhibited the most potency against *Bacillus cereus*, *Bacillus subtilis*, *Staphylococcus aureus*, *Escherichia coli* and *Pseudomonas aeruginosa*. Analogues **5-02**, **5-07**, **5-12**, **5-15**, **5-19**, **5-20** and **5-25** could effectively inhibit the spore germination of *Botrytis cinerea*. The relationship between structure and their antimicrobial activity (SAR) has also been discussed according to aliphatic acids and aromatic acids derivatives, respectively. This implied that the *N*-acylated derivatives of 5-methyl-benzimidazolone might be potential antimicrobial agents.

## 1. Introduction

Benzimidazolone and their derivatives are useful intermediates in the production of pharmaceuticals and pesticides [1]. For example, benzimidazolone carboxylic acids derivatives are potential therapeutic agents of selective peroxisome proliferator-activated receptor *γ* modulators (SPPARγMs) and are useful for the treatment of type II diabetes mellitus (T2DM) [2]. Additionally, benzoxazolone derivatives have been found to be antagonists of opioid receptor-like 1 (NOP), *N*-methyl-D-aspartate (NMDA) and mGlu5 receptors [3–7] and *Mycobacterium tuberculosis* enzyme, non-nucleoside reverse transcriptase, D-amino acid oxidase (DAAO) and heat shock protein 90 (HSP90) inhibitors [8–11]. There are also several reports on the antimicrobial activity of benzimidazolones. For instance, it has been reported in recent literature that benzimidazolones, which contain a sugar residue or piperidine ring, exhibited antibacterial activities to several strains of Gram-positive or Gram-negative bacteria [12,13].

**Figure 1:**
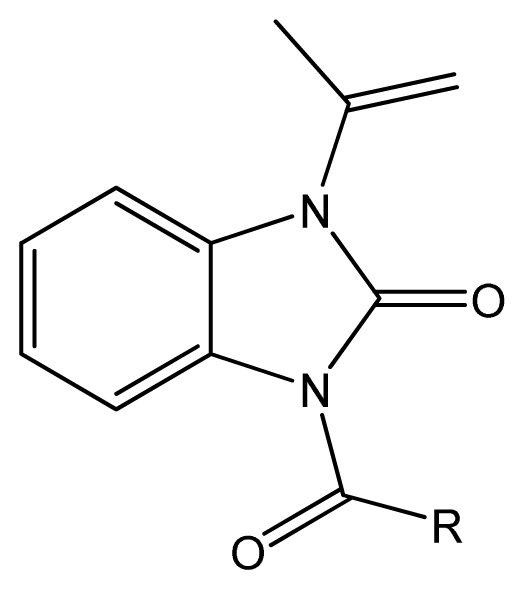
*N*-acylated isopropenyl-benzimidazolones.

In previous investigation, we synthesized a series of *N*-acylated isopropenyl-benzimidazolones (Figure 1), in which several derivatives exhibited strong antifungal activity against *Botrytis cinerea*[14–16]. To better reveal the relationship between the structures and their antimicrobial activities of benzimidazolones, especially the influence of introducing substituted groups to the benzene ring, a series of *N*-acylated analogues of 1-isopropyl-3-acyl-5-methyl-benzimidazolone were synthesized and evaluated for antimicrobial activity herein.

## 2. Results and Discussion

### 2.1. Chemistry

**Scheme 1:**
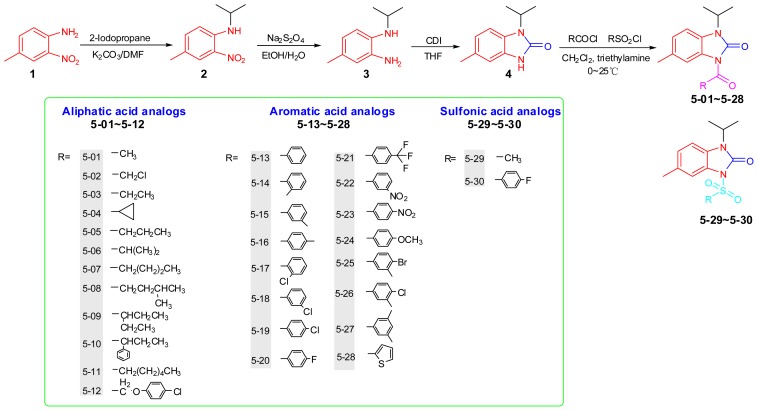
Synthetic route of the title compounds.

The basic strategy for the synthesis of the target substances involves the *N*-acylation reaction of 1-isopropyl-5-methyl-1*H*-benzo[*d*]imidazol-2(3*H*)-one with acyl chlorides. With the aim of studying the structure–activity relationship (SAR, three types of 1-isopropyl-3-acyl-5-methyl-benzimi-dazolone analogues were prepared by introducing a variety of aliphatic acids, aromatic acids and sulfonic acids to the heterocyclic scaffold. Starting from 4-methyl-2-nitroaniline (**1**), the synthetic route of 1-isopropyl-3-acyl-5-methyl-1*H*-benzo[*d*]imidazol-2(3*H*)-one (**5-01**~**5-30**) is shown in Scheme 1.

1-Isopropyl-4-methyl-2-nitroaniline (**2**) was obtained by introducing the isopropyl group via *N*-alkylation reaction in *N*, *N*-dimethylformamide (DMF) [17]. After the nitro group was reduced with Na_2_S_2_O_4_ to afford **3**[18] and subsequent cyclization with CDI [19], 1-isopropyl-5-methyl-1*H*-benzo[*d*] imidazol-2(3*H*)-one (**4**) was finally obtained. Title compounds were prepared by *N*-acylation of **4** with corresponding acid chlorides, as depicted in Scheme 1. The chemical structures of the compounds have been elucidated by ^1^H-NMR, ^13^C-NMR and elemental analysis.

### 2.2. Antimicrobial Activities of 1-isopropyl-3-acyl-5-methyl-benzimidazolones

Antibacterial activity of the title compounds against standard bacterial strains was evaluated by the methods of paper disc-diffusion and micro-broth dilution, and antifungal activity against *B. cinerea* was evaluated by spore germination assay. The antimicrobial test results indicated that sulfonic acids and most of the carboxylic acids derivatives were not effective against tested strains, and the results are summarized in Tables 1 and 2. To make a judgment of their antimicrobial potency, minimum inhibitory concentration (MIC) and 50% effect concentration (EC_50_) values of several compounds with high activity were further determined; ampicillin and azoxystrobin were used as the positive control, respectively.

#### Antibacterial activity

Among aliphatic acids derivatives, **5-02** and **5-07** exhibited antibacterial activity against both Gram-positive and Gram-negative bacteria, as well as **5-12** had moderate activity against two Gram-positive strains (Table 1). The MIC values of these three compounds were determined by the method of micro-broth dilution, and the results indicated that **5-07** (pentanoic acid derivative) had the most strong inhibitory action; its MICs against *B. cereus*, *B. subtilis*, *S. aureus*, *E. coli* and *P. aeruginosa* were 25.0, 12.5, 50.0, 50.0 and 100.0 μg/mL, respectively (Table 2). For aromatic acids derivatives, **5-15**, **5-19**, **5-20**, **5-25** and **5-26** exhibited antibacterial activity against both Gram-positive and Gram-negative bacteria, as well as **5-14**, **5-17**, **5-21** and **5-22** had moderate activity against several Gram-positive strains (Table 1). Further determination of MICs revealed that **5-19** (4-chloro-benzoic acid derivative) was the most active compound; its MICs against *B. cereus*, *B. subtilis*, *S. aureus*, *E. coli* and *P. aeruginosa* were 6.25, 12.5, 12.5, 50.0 and 100.0 μg/mL, respectively (Table 2). For sulfonic acid derivatives, both compounds (**5-29**, **5-30**) did not exhibit inhibitory action against the tested bacteria. Another phenomenon we noted throughout the antibacterial tests is that Gram-positive bacteria are more sensitive to the active compounds than Gram-negative strains.

Analysis of the relationship between structure and antibacterial activity of aliphatic acid derivatives implies that a steric effect may play a crucial role for the activity. If the introduced groups are similar in physicochemical properties, the derivatives should exhibit equivalent bioactivities, whereas the activity of the pentanoic acid derivative was much stronger than its homologue. This phenomena could not be interpreted by electronic effect, and it may be ascribed to the size of the substitutions. In other words, the activity was strongly influenced by the length of carbon chain, and the optimal length was five carbons (**5-07**); the introduction of other short-chain or long-chain aliphatic acids was not beneficial to the activity. It should be noted that **5-02** and **5-12**, two substituted aliphatic acid derivatives, also exhibited antibacterial activity, even though their activity was dramatically lower than **5-07,** and this result could not be interpreted by the steric effect. Comparing the structures of **5-02** and **5-12** to that of **5-10**, another substituted aliphatic acid derivative, the difference between them was the presence of a high electronegative atom (O or Cl) connected to the α-carbon. It revealed that the antibacterial activity of substituted aliphatic acid derivatives might be influenced by the electronic effect of substituted groups at the α-carbon.

Based on the data from the antibacterial tests of aromatic acid derivatives, the following observations can be made. Compounds **5-15**, **5-19**, **5-20**, **5-25** and **5-26** exhibited antibacterial activity against all tested strains, which indicated that introducing a methyl group at the three-position of benzoic acids or halogen at the four-position or both of them at the same time was beneficial to activity. More importantly, the toxicity of **5-19**, 4-chlorobenzoic acid derivative, against tested bacteria was dramatically higher than its analogues, especially for **5-20**, 4-fluorobenzoic acid derivative, in spite of similar structural characteristic. It implies that the activity requires harsh chemical conditions.

#### Antifungal activity

The results of antifungal activities indicated that chloroacetic acid (**5-02**), pentanoic acid (**5-07**), 4-chlorophenoxy acetic acid (**5-12**), 3-methy- (**5-15**), 4-chloro- (**5-19**), 4-fluoro- (**5-20**) and 4-bromo-3-methylbenzoic acid (**5-25**) derivatives of 1-isopropyl-3-acyl-5-methyl-benzimidazol-one exhibited antifungal activity against *B. cinerea*; their inhibition rates of spore germination were all above 70% at the concentration of 25 μg/mL. Their EC_50_ values against *B. cinerea* were further determined as 21.07, 17.62, 10.68, 19.75, 14.23, 17.33 and 16.39 μg/mL, respectively. For the aliphatic acids derivatives, **5-02**, **5-07** and **5-12** had stronger antifungal activity than their analogues, and they were also the most potent compounds in the test of antibacterial screening. A variation observed was that these three compounds exhibited similar antifungal activity, whereas the antibacterial toxicity of **5-07** was higher than **5-02** and **5-12**, more obviously. For aromatic acid derivatives, the key structural characteristics of active compounds were ascribed to a methyl group at the three-position of benzoic acid or halogen at the four-position of benzoic acid or both of them at the same time, respectively. Generally, although there were differences in toxicity with the same class of chemicals, a similar tendency of SAR is observed in the screening of both antibacterial and antifungal activity.

By comparison of the results of our previous investigation, we noted that the introduction of a methyl group at the five-position of the benzimidazolone scaffold could affect the activity of the derivatives obviously. For example, the antifungal toxicity of the 2-chloro-benzoic acid derivative of 1-isopropenyl-benzimi-dazolone was higher than the 4-chloro-benzoic acid derivative, whereas the contrary results were observed in their counterparts of 1-isopropyl-5-methyl-benzimidazolone [14]. This difference might be attributed to the influence of methyl substituted at the five-position. It implies that the substituted group at aromatic ring of the scaffold plays an important role for antimicrobial activity. In general, this is just a preliminary result. Introducing methyl group to the four-, six- or seven-position or introducing other substitutions to the scaffold may give different results. To further understand the relationship between structures and activity, more derivatives are needed to be investigated in the future.

## 3. Experimental Section

### 3.1. General Experimental Procedures

^1^H NMR and ^13^C NMR spectra were measured on a Bruker AVANCE III NMR spectrometer (Bruker Daltonics Inc.), using tetramethylsilane (Me_4_Si) as an internal standard (^1^H at 500 MHz, ^13^C at 125 MHz, respectively). Elemental analyses were carried out on an Elementar Vairo EL analyzer (Elementar Analysensysteme Co., Germany). Reaction progress was monitored by thin-layer chromatography (TLC) on silica gel GF_254_ with detection by ultraviolet. Silica gel (200~300 mesh, Qingdao Chemical Co., China) was used for column chromatography. The purities of tested compounds were determined to be greater than 95%, based on reverse-phase HPLC analysis (Hypersil BDS C18, 4.6 mm × 250 mm, 5 μm) under the following conditions: flow rate, 1.0 mL/min; mobile phase, methanol-water at the ratio of 7:3 (v/v); detection, λ = 235 nm.

### 3.2. Synthetic Procedures

#### 3.2.1. Synthesis of N-isopropyl-4-methyl-2-nitroaniline (**2**)

To a solution of **1** (4.56 g, 30 mmol) in 50 mL of dimethylformamide (DMF) was added K_2_CO_3_ (8.28 g, 60 mmol) and 2-iodopropane (6.80 g, 40 mmol). The mixture was stirred at room temperature for 24 h. TLC analysis showed the reaction complete. The reaction solution was diluted with 100 mL of ice water and extracted with ethyl acetate (50 mL × 3). The organic layers were combined, washed by brine and dried over anhydrous sodium sulfate. The solvent was evaporated under vacuum to afford the crude product **2**, which was used in subsequent steps without purification.

#### 3.2.2. Synthesis of N^1^-isopropyl-4-methylbenzene-1,2-diamine (**3**)

To compound **2** (2.91 g, 15 mmol) was added ethanol (20 mL) and water (40 mL) at room temperature. The mixture was heated to reflux, and then, the solution of Na_2_S_2_O_4_ (7.83 g, 45 mmol) in water (100 mL) was added dropwise. After stirring for 0.5 h, the mixture was cooled to room temperature and extracted with ethyl acetate (50 mL × 3). The combined organic layers were washed with brine, dried over Na_2_SO_4_ and concentrated in vacuum. The concentrated mixture was subjected to a silica gel column and eluted by the mixture of petroleum ether and ethyl acetate (2/1, v/v) to afford compound **3** as a thick liquid (1.97 g, yield 40%). ^1^H NMR (CDCl_3_): 1.46 (d, *J* = 8.0 Hz, 6H, 2CH_3_), 2.32 (s, 3H, CH_3_), 4.68 (m, 1H, CH), 6.87 (d, *J* = 8.5 Hz, 1H, Ph), 7.29 (d, *J* = 8.5 Hz, 1H, Ph), 7.96 (s, 1H, Ph), 8.11 (s, 1H, NH). HR-ESI/MS: m/z calculated (calcd.) for C_10_H_17_N_2_ ([M + H]^+^) 165.1392, found 165.1398.

#### 3.2.3. Synthesis of 1-isopropyl-5-methyl-1H-benzo[d]imidazol-2(3H)-one (**4**)

To a solution of **3** (0.82 g, 5 mmol) in anhydrous tetrahydrofuran (15 mL) was added *N*, *N*-carbonyl-diimidazole (CDI, 1.62 g, 10 mmol). The reaction mixture was stirred for 20 h at room temperature. The solvent was evaporated in vacuum. The residue was diluted with 10 mL of water, extracted with EtOAc. The combined organic layers were washed with brine, dried over Na_2_SO_4_, filtered and concentrated to give a brown solid. Purification by silica gel column chromatography gave compound **4** (0.68 g, yield 71%) as a white solid, mp, 153–155 °C. ^1^H NMR (CDCl_3_): 1.61 (d, *J* = 7.5 Hz, 6H, 2CH_3_), 2.45 (s, 3H, CH_3_), 4.71 (m, 1H, CH), 7.05 (s, 2H, Ph), 8.10 (s, 1H, Ph), 10.64 (s, 1H, NH). Anal. Calcd for C_11_H_14_N_2_O: C, 69.45; H, 7.42; N, 14.73. Found: C, 69.57; H, 7.44; N, 14.79.

#### 3.2.4. Synthesis of 3-acyl-1-isopropyl-5-methyl-benzimidazolones (**5-01**~**5-30**)

To a solution of **4** (95 mg, 5 mmol) in dichloromethane (10 mL) was added triethylamine (1.2 mL). Chloride (6 mmol) in dichloromethane (2 mL) was added dropwise in an ice-water bath, and the resulting mixture was stirred for 5 h at room temperature. To the reaction solution was added saturated NaHCO_3_ (20 mL), and the mixture was stirred for 30 min at room temperature. After extraction by dichloromethane, the organic extracts were combined, washed with water and brine, dried over Na_2_SO_4_ and concentrated in vacuum. The concentrated mixture was subjected to a silica gel column and eluted by the mixture of petroleum ether and ethyl acetate (5/1, v/v) to give target compounds. The structures of the title compounds were characterized by ^1^H NMR, ^13^C NMR and elemental analysis, and their data are listed below.

*3-acetyl-1-isopropyl-5-methyl-1H-benzo[d]imidazol-2(3H)-one* (**5-01**): white crystalline solid; yield, 68%, mp, 146–148 °C. ^1^H NMR (CDCl_3_): 1.54 (d, *J* = 8.5 Hz, 6H, 2CH_3_), 2.39 (s, 3H, CH_3_), 2.75 (s, 3H, CH_3_), 4.67 (m, 1H, CH), 7.00 (s, 2H, Ph), 8.08 (s, 1H, Ph). ^13^C NMR (CDCl_3_): 19.79, 21.44, 25.82, 45.25, 108.40, 116.51, 124.70, 126.47, 126.52, 132.08, 151.84, 170.87. Analysis (anal.) calcd. for C_13_H_16_N_2_O_2_: C, 67.22; H, 6.94; N, 12.06. Found: C, 67.20; H, 6.93; N, 12.02.

*3-(2-chloroacetyl)-1-isopropyl-5-methyl-1H-benzo[d]imidazol-2(3H)-one* (**5-02**): white crystalline solid; yield, 55%, mp, 158–160 °C. ^1^H NMR (CDCl_3_): 1.54 (d, *J* = 8.5 Hz, 6H, 2CH_3_), 2.40 (s, 3H, CH_3_), 4.65 (m, 1H, CH), 4.96 (s, 2H, CH_2_), 7.01-7.06 (m, 2H, Ph), 8.09 (s, 1H, Ph). ^13^C NMR (CDCl_3_): 19.78, 21.45, 45.28, 45.57, 108.77, 116.51, 125.35, 126.02, 126.85, 132.61, 151.30, 166.28. Anal. calcd. for C_13_H_15_ClN_2_O_2_: C, 58.54; H, 5.67; N, 10.50. Found: C, 58.02; H, 5.63; N, 10.72.

*1-isopropyl-5-methyl-3-propionyl-1H-benzo[d]imidazol-2(3H)-one* (**5-03**): white crystalline solid; yield, 75%, mp, 119–120 °C. ^1^H NMR (CDCl_3_): 1.27 (t, *J* = 9.0 Hz, 3H, CH_3_), 1.53 (d, *J* = 8.5 Hz, 6H, 2CH_3_), 2.39 (s, 3H, CH_3_), 3.18 (q, *J* = 9.0 Hz, 2H, CH_2_), 4.67 (m, 1H, CH), 7.00 (s, 2H, Ph), 8.11 (s, 1H, Ph). ^13^C NMR (CDCl_3_): 8.34, 19.80, 21.44, 31.00, 45.18, 108.36, 116.52, 124.54, 126.54, 126.65, 132.01, 151.74, 174.86. Anal. calcd. for C_14_H_18_N_2_O_2_: C, 68.27; H, 7.37; N, 11.37. Found: C, 68.25; H, 7.33; N, 11.32.

*3-(cyclopropanecarbonyl)-1-isopropyl-5-methyl-1H-benzo[d]imidazol-2(3H)-one* (**5-04**): white crystalline solid; yield, 61%, mp, 82–84 °C. ^1^H NMR (CDCl_3_): 1.08 (q, *J* = 9.0 Hz, 2H, CH_2_), 1.27 (q, *J* = 9.0 Hz, 2H, CH_2_), 1.55 (d, *J* = 8.5 Hz, 6H, 2CH_3_), 2.37 (s, 3H, CH_3_), 3.55 (m, 1H, CH), 4.67 (m, 1H, CH), 7.00 (s, 2H, Ph), 8.04 (s, 1H, Ph). ^13^C NMR (CDCl_3_): 11.16, 14.00, 19.80, 21.43, 45.25, 108.333, 116.50, 124.51, 126.22, 126.87, 132.00, 152.26, 175.07. Anal. calcd. for C_15_H_18_N_2_O_2_: C, 69.74; H, 7.02; N, 10.84. Found: C, 69.75; H, 7.03; N, 10.82.

*3-butyryl-1-isopropyl-5-methyl-1H-benzo[d]imidazol-2(3H)-one* (**5-05**): white crystalline solid; yield, 72%, mp, 114–115 °C. ^1^H NMR (CDCl_3_): 1.05 (t, *J* = 9.0 Hz, 3H, CH_3_), 1.53 (d, *J* = 8.5 Hz, 6H, 2CH_3_), 1.80 (m, 2H, CH_2_), 2.39 (s, 3H, CH_3_), 3.15 (t, *J* = 9.0 Hz, 2H, CH_2_), 4.67 (m, 1H, CH), 6.99 (s, 2H, Ph), 8.11 (s, 1H, Ph). ^13^C NMR (CDCl_3_): 13.75, 17.69, 19.79, 21.44, 39.30, 45.20, 108.34, 116.55, 124.55, 126.53, 126.66, 132.00, 151.74, 173.99. Anal. calcd. for C_15_H_20_N_2_O_2_: C, 69.20; H, 7.74; N, 10.76. Found: C, 69.25; H, 7.73; N, 10.72.

*3-isobutyryl-1-isopropyl-5-methyl-1H-benzo[d]imidazol-2(3H)-one* (**5-06**): white crystalline solid; yield, 52%, mp, 55–57 °C. ^1^H NMR (CDCl_3_): 1.28 (d, *J* = 8.5 Hz, 6H, 2CH_3_), 1.53 (d, *J* = 8.5 Hz, 6H, 2CH_3_), 2.38 (s, 3H, CH_3_), 4.11 (m, 1H, CH), 4.67 (m, 1H, CH), 7.00 (s, 2H, Ph), 8.10 (s, 1H, Ph). ^13^C NMR (CDCl_3_): 19.00, 19.80, 21.44, 34.25, 45.22, 108.35, 116.71, 124.53, 126.59, 126.96, 132.00, 151.38, 178.58. Anal. calcd. for C_15_H_20_N_2_O_2_: C, 69.20; H, 7.74; N, 10.76. Found: C, 69.15; H, 7.78; N, 10.79.

*1-isopropyl-5-methyl-3-pentanoyl-1H-benzo[d]imidazol-2(3H)-one* (**5-07**): white crystalline solid; yield, 82%, mp, 58–60 °C. ^1^H NMR (CDCl_3_): 0.97 (t, *J* = 9.0 Hz, 3H, CH_3_), 1.44 (m, 2H, CH_2_), 1.53 (d, *J* = 8.5 Hz, 6H, 2CH_3_), 1.77 (m, 2H, CH_2_), 2.39 (s, 3H, CH_3_), 3.17 (t, *J* = 8.5 Hz, 2H, CH_2_), 4.67 (m, 1H, CH), 7.00 (s, 2H, Ph), 8.10 (s, 1H, Ph). ^13^C NMR (CDCl_3_): 13.93, 19.80, 20.24, 21.44, 26.33, 37.16, 45.20, 108.36, 116.55, 124.54, 126.52, 126.69, 132.01, 151.73, 174.19. Anal. calcd. for C_16_H_22_N_2_O_2_: C, 70.04; H, 8.08; N, 10.21. Found: C, 70.09; H, 8.10; N, 10.29.

*1-isopropyl-5-methyl-3-(4-methylpentanoyl)-1H-benzo[d]imidazol-2(3H)-one* (**5-08**): white solid; yield, 42%, mp, 70–72 °C. ^1^H NMR (CDCl_3_): 0.96 (d, *J* = 8.0 Hz, 6H, 2CH_3_), 1.53 (d, *J* = 8.5 Hz, 6H, 2CH_3_), 1.70 (m, 3H, CH, CH_2_), 2.39 (s, 3H, CH_3_), 3.17 (t, *J* = 8.5 Hz, 1H, CH), 4.67 (m, 1H, CH), 7.00 (s, 2H, Ph), 8.10 (s, 1H, Ph). ^13^C NMR (CDCl_3_): 19.81, 21.44, 22.45, 27.70, 33.04, 35.51, 45.18, 108.38, 116.56, 124.54, 126.52, 127.55, 132.00, 151.76, 174.45. Anal. calcd. for C_17_H_24_N_2_O_2_: C, 70.80; H, 8.39; N, 9.71. Found: C, 70.89; H, 8.30; N, 9.79.

*3-(2-ethylbutanoyl)-1-isopropyl-5-methyl-1H-benzo[d]imidazol-2(3H)-one* (**5-09**): white crystalline solid; yield, 55%, mp, 75–76 °C. ^1^H NMR (CDCl_3_): 0.96 (t, *J* = 8.5 Hz, 6H, 2CH_3_), 1.53 (d, *J* = 8.5 Hz, 6H, 2CH_3_), 1.65 (m, 2H, CH_2_), 1.86 (m, 2H, CH_2_), 2.39 (s, 3H, CH_3_), 4.00 (m, 1H, CH), 4.67 (m, 1H, CH), 7.00 (s, 2H, Ph), 8.14 (s, 1H, Ph). ^13^C NMR (CDCl_3_): 11.58, 19.82, 21.44, 24.48, 45.16, 47.28, 108.41, 116.71, 124.53, 126.47, 126.95, 131.99, 151.60, 177.50. Anal. calcd. for C_17_H_24_N_2_O_2_: C, 70.80; H, 8.39; N, 9.71. Found: C, 70.69; H, 8.40; N, 9.69.

*1-isopropyl-5-methyl-3-(2-phenylbutanoyl)-1H-benzo[d]imidazol-2(3H)-one* (**5-10**): white solid; yield, 45%, mp, 65–67 °C. ^1^H NMR (CDCl_3_): 0.94 (t, *J* = 8.5 Hz, 3H, CH_3_), 1.49 (d, *J* = 8.5 Hz, 6H, 2CH_3_), 1.91, 2.25 (m, 2H, CH_2_), 2.37 (s, 3H, CH_3_), 4.66 (m, 1H, CH), 5.30 (t, *J* = 9.0 Hz, 1H, CH), 6.96 (s, 2H, Ph), 7.31 (m, 3H, Ph), 7.46 (m, 2H, Ph), 8.14 (s, 1H, Ph). ^13^C NMR (CDCl_3_): 12.21, 19.74, 19.77, 21.41, 27.49, 45.14, 52.05, 108.43, 116.71, 124.63, 126.40, 126.97, 127.09, 128.08, 128.44, 128.57, 128.83, 131.99, 138.95, 151.40, 174.87. Anal. calcd. for C_21_H_24_N_2_O_2_: C, 74.97; H, 7.19; N, 8.33. Found: C, 74.89; H, 7.20; N, 8.39.

*3-heptanoyl-1-isopropyl-5-methyl-1H-benzo[d]imidazol-2(3H)-one* (**5-11**): white crystalline solid; yield, 74%, mp, 55–57 °C. ^1^H NMR (CDCl_3_): 0.89 (t, *J* = 9.0 Hz, 3H, CH_3_), 1.32~1.42 (m, 6H, 3CH_2_), 1.53 (d, *J* = 8.5 Hz, 6H, 2CH_3_), 1.77 (m, 2H, CH_2_), 2.39 (s, 3H, CH_3_), 3.17 (t, *J* = 8.5 Hz, 2H, CH_2_), 4.67 (m, 1H, CH), 6.99 (s, 2H, Ph), 8.11 (s, 1H, Ph). ^13^C NMR (CDCl_3_): 14.05, 19.80, 21.44, 22.57, 24.19, 28.87, 31.64, 37.42, 45.19, 108.34, 116.56, 124.54, 126.52, 126.68, 132.00, 151.73, 174.20. Anal. calcd. for C_18_H_26_N_2_O_2_: C, 71.49; H, 8.67; N, 9.26. Found: C, 71.38; H, 8.66; N, 9.29.

*3-(2-(4-chlorophenoxy)acetyl)-1-isopropyl-5-methyl-1H-benzo[d]imidazol-2(3H)-one* (**5-12**): white crystalline solid; yield, 65%, mp, 69–71 °C. ^1^H NMR (CDCl_3_): 1.55 (d, *J* = 8.5 Hz, 6H, 2CH_3_), 2.38 (s, 3H, CH_3_), 4.60 (m, 1H, CH), 5.39 (s, 2H, CH_2_), 6.92 (s, 2H, Ph), 7.04 (m, 2H, Ph), 7.25 (m, 2H, Ph), 8.08 (s, 1H, Ph). ^13^C NMR (CDCl_3_): 19.80, 21.41, 45.56, 68.55, 108.77, 116.16, 116.44, 125.28, 125.76, 126.50, 126.79, 129.43, 132.62, 151.48, 156.65, 168.43. Anal. calcd. for C_19_H_19_ClN_2_O_3_: C, 63.60; H, 5.34; N, 7.81. Found: C, 63.55; H, 5.37; N, 7.80.

*3-benzoyl-1-isopropyl-5-methyl-1H-benzo[d]imidazol-2(3H)-one* (**5-13**): yellow solid; yield, 77%, mp, 52–54 °C. ^1^H NMR (CDCl_3_): 1.54 (d, *J* = 8.5 Hz, 6H, 2CH_3_), 2.38 (s, 3H, CH_3_), 4.74 (m, 1H, CH), 7.00 (s, 2H, Ph), 7.47 (m, 3H, Ph), 7.60 (m, 2H, Ph), 8.11 (s, 1H, Ph). ^13^C NMR (CDCl_3_): 20.26, 21.30, 44.93, 109.31, 115.36, 124.68, 126.58, 127.03, 128.02, 128.13, 128.44, 129.27, 129.82, 132.55, 138.95, 151.43, 171.65. Anal. calcd. for C_18_H_18_N_2_O_2_: C, 73.45; H, 6.16; N, 9.52. Found: C, 73.49; H, 6.20; N, 9.49.

*1-isopropyl-5-methyl-3-(2-methylbenzoyl)-1H-benzo[d]imidazol-2(3H)-one* (**5-14**): white solid; yield, 77%, mp, 95–96 °C. ^1^H NMR (CDCl_3_): 1.47 (d, *J* = 8.5 Hz, 6H, 2CH_3_), 2.37 (s, 3H, CH_3_), 2.43 (s, 3H, CH_3_), 4.60 (m, 1H, CH), 7.01 (s, 2H, Ph), 7.45~7.50 (m, 4H, Ph), 8.11 (s, 1H, Ph). ^13^C NMR (CDCl_3_): 18.12, 19.75, 21.41, 45.13, 108.31, 116.36, 124.68, 126.41, 126.93, 128.06, 128.33, 128.54, 128.87, 129.82, 132.15, 138.88, 151.53, 172.67. Anal. calcd. for C_19_H_20_N_2_O_2_: C, 74.00; H, 6.54; N, 9.08. Found: C, 74.09; H, 6.50; N, 9.09.

*1-isopropyl-5-methyl-3-(3-methylbenzoyl)-1H-benzo[d]imidazol-2(3H)-one* (**5-15**): white solid; yield, 77%, mp, 103–105 °C. ^1^H NMR (CDCl_3_): 1.47 (d, *J* = 8.5 Hz, 6H, 2CH_3_), 2.37 (s, 3H, CH_3_), 2.43 (s, 3H, CH_3_), 4.60 (m, 1H, CH), 7.01 (s, 2H, Ph), 7.45~7.50 (m, 4H, Ph), 8.11 (s, 1H, Ph). ^13^C NMR (CDCl_3_): 18.12, 19.75, 21.41, 45.13, 108.31, 116.36, 124.68, 126.41, 126.93, 128.06, 128.33, 128.54, 128.87, 129.82, 132.15, 138.88, 151.53, 172.67. Anal. calcd. for C_19_H_20_N_2_O_2_: C, 74.00; H, 6.54; N, 9.08. Found: C, 74.07; H, 6.59; N, 9.11.

*1-isopropyl-5-methyl-3-(4-methylbenzoyl)-1H-benzo[d]imidazol-2(3H)-one* (**5-16**): white crystalline solid; yield, 77%, mp, 123–125 °C. ^1^H NMR (CDCl_3_): 1.47 (d, *J* = 8.5 Hz, 6H, 2CH_3_), 2.37 (s, 3H, CH_3_), 2.43 (s, 3H, CH_3_), 4.60 (m, 1H, CH), 7.01 (s, 2H, Ph), 7.45~7.50 (m, 4H, Ph), 8.11 (s, 1H, Ph). ^13^C NMR (CDCl_3_): 18.12, 19.75, 21.41, 45.13, 108.31, 116.36, 124.68, 126.41, 126.93, 128.06, 128.33, 128.54, 128.87, 129.82, 132.15, 138.88, 151.53, 172.67. Anal. calcd. for C_19_H_20_N_2_O_2_: C, 74.00; H, 6.54; N, 9.08. Found: C, 73.97; H, 6.49; N, 9.00.

*3-(2-chlorobenzoyl)-1-isopropyl-5-methyl-1H-benzo[d]imidazol-2(3H)-one* (**5-17**): white crystalline solid; yield, 74%, mp, 74–76 °C. ^1^H NMR (CDCl_3_): 1.48 (d, *J* = 8.5 Hz, 6H, 2CH_3_), 2.44 (s, 3H, CH_3_), 4.58 (m, 1H, CH), 7.01 (s, 2H, Ph), 7.45~7.55 (m, 4H, Ph), 8.11 (s, 1H, Ph). ^13^C NMR (CDCl_3_): 19.73, 21.43, 45.37, 108.83, 116.10, 125.22, 126.67, 126.96, 127.04, 128.25, 129.38, 130.75, 131.24, 132.34, 133.36, 150.75, 166.60. Anal. calcd. for C_18_H_17_ClN_2_O_2_: C, 65.75; H, 5.21; N, 8.52. Found: C, 65.77; H, 5.29; N, 8.56.

*3-(3-chlorobenzoyl)-1-isopropyl-5-methyl-1H-benzo[d]imidazol-2(3H)-one* (**5-18**): white crystalline solid; yield, 71%, mp, 138–140 °C. ^1^H NMR (CDCl_3_): 1.55 (d, *J* = 8.5 Hz, 6H, 2CH_3_), 2.39 (s, 3H, CH_3_), 4.72 (m, 1H, CH), 7.01 (s, 2H, Ph), 7.40~7.60 (m, 4H, Ph), 8.11 (s, 1H, Ph). ^13^C NMR (CDCl_3_): 19.73, 21.43, 45.37, 108.83, 116.10, 125.22, 126.67, 126.96, 127.04, 128.25, 129.38, 130.75, 131.24, 132.34, 133.36, 150.75, 166.60. Anal. calcd. for C_18_H_17_ClN_2_O_2_: C, 65.75; H, 5.21; N, 8.52. Found: C, 65.70; H, 5.20; N, 8.55.

*3-(4-chlorobenzoyl)-1-isopropyl-5-methyl-1H-benzo[d]imidazol-2(3H)-one* (**5-19**): white crystalline solid; yield, 71%, mp, 178–180 °C. ^1^H NMR (CDCl_3_): 1.52 (d, *J* = 8.5 Hz, 6H, 2CH_3_), 2.42 (s, 3H, CH_3_), 4.59 (m, 1H, CH), 7.01 (s, 2H, Ph), 7.40~7.60 (m, 4H, Ph), 8.11 (s, 1H, Ph). ^13^C NMR (CDCl_3_): 19.73, 21.45, 45.33, 108.73, 116.18, 125.32, 126.69, 126.96, 127.54, 128.55, 129.18, 130.11, 131.65, 132.44, 133.76, 151.05, 167.60. Anal. calcd. for C_18_H_17_ClN_2_O_2_: C, 65.75; H, 5.21; N, 8.52. Found: C, 65.70; H, 5.27; N, 8.50.

*3-(4-fluorobenzoyl)-1-isopropyl-5-methyl-1H-benzo[d]imidazol-2(3H)-one* (**5-20**): white solid; yield, 70%, mp, 58–60 °C. ^1^H NMR (CDCl_3_): 1.53 (d, *J* = 8.5 Hz, 6H, 2CH_3_), 2.39 (s, 3H, CH_3_), 4.59 (m, 1H, CH), 7.01 (s, 2H, Ph), 7.40~7.60 (m, 4H, Ph), 8.11 (s, 1H, Ph). ^13^C NMR (CDCl_3_): 19.75, 21.40, 45.03, 108.78, 116.10(d, *J* = 22.5 Hz), 125.30, 126.59, 128.04, 129.12(d, *J* = 8.3 Hz), 130.51, 132.49, 133.79, 150.95, 160.38 (d, *J* = 243.4 Hz), 167.60. Anal. calcd. for C_18_H_17_FN_2_O_2_: C, 69.22; H, 5.49; N, 8.97. Found: C, 69.25; H, 5.47; N, 8.90.

*1-isopropyl-5-methyl-3-(4-(trifluoromethyl)benzoyl)-1H-benzo[d]imidazol-2(3H)-one* (**5-21**): yellow solid; yield, 61%, mp, 100–102 °C. ^1^H NMR (CDCl_3_): 1.52 (d, *J* = 8.5 Hz, 6H, 2CH_3_), 2.43 (s, 3H, CH_3_), 4.60 (m, 1H, CH), 7.07 (s, 2H, Ph), 7.73~7.90 (m, 4H, Ph), 8.31 (s, 1H, Ph). ^13^C NMR (CDCl_3_): 19.78, 21.44, 45.53, 108.99, 115.68, 123.61(q, *J* = 272.5 Hz), 125.16, 125.74 (q, *J* = 3.8 Hz), 126.62, 127.14, 128.62, 132.36, 134.42(q, *J* = 32.7 Hz), 139.75(q, *J* = 1.2 Hz,), 151.19, 171.13. Anal. calcd. for C_19_H_17_F_3_N_2_O_2_: C, 62.98; H, 4.73; N, 7.73. Found: C, 62.90; H, 4.77; N, 7.70.

*1-isopropyl-5-methyl-3-(3-nitrobenzoyl)-1H-benzo[d]imidazol-2(3H)-one* (**5-22**): yellow crystalline solid; yield, 82%, mp, 151–153 °C. ^1^H NMR (CDCl_3_): 1.52 (d, *J* = 8.5 Hz, 6H, 2CH_3_), 2.43 (s, 3H, CH_3_), 4.57 (m, 1H, CH), 7.07 (s, 2H, Ph), 7.66 (t, *J* = 8.0 Hz, 1H, Ph), 7.90 (s, 1H, Ph), 8.02 (dd, *J* = 8.0 Hz, 1H, Ph), 8.42 (dd, *J* = 8.0 Hz, 1H, Ph), 8.56 (s, 1H, Ph). ^13^C NMR (CDCl_3_): 19.75, 21.43, 45.65, 109.07, 115.73, 124.21, 125.38, 126.44, 126.48, 127.13, 129.06, 132.46, 134.64, 135.94, 147.74, 151.12, 166.91. Anal. calcd. for C_18_H_17_N_3_O_4_: C, 63.71; H, 5.05; N, 12.38. Found: C, 63.76; H, 5.07; N, 12.31.

*1-isopropyl-5-methyl-3-(4-nitrobenzoyl)-1H-benzo[d]imidazol-2(3H)-one* (**5-23**): yellow crystalline solid; yield, 79%, mp, 140–142 °C. ^1^H NMR (CDCl_3_): 1.52 (d, *J* = 8.5 Hz, 6H, 2CH_3_), 2.43 (s, 3H, CH_3_), 4.57 (m, 1H, CH), 7.07 (s, 2H, Ph), 7.83 (d, *J* = 11.0 Hz, 2H, Ph), 7.94 (s, 1H, Ph), 8.32 (d, *J* = 11.0 Hz, 2H, Ph). ^13^C NMR (CDCl_3_): 19.74, 21.44, 45.65, 109.06, 115.82, 123.26, 125.44, 126.36, 127.16, 129.60, 132.52, 140.24, 149.51, 151.06, 167.37. Anal. calcd. for C_18_H_17_N_3_O_4_: C, 63.71; H, 5.05; N, 12.38. Found: C, 63.68; H, 4.99; N, 12.30.

*1-isopropyl-3-(4-methoxybenzoyl)-5-methyl-1H-benzo[d]imidazol-2(3H)-one* (**5-24**): white solid; yield, 71%, mp, 65–67 °C. ^1^H NMR (CDCl_3_): 1.52 (d, *J* = 8.5 Hz, 6H, 2CH_3_), 2.40 (s, 3H, CH_3_), 3.87 (s, 3H, CH_3_), 4.62 (m, 1H, CH), 6.93~7.05 (m, 3H, Ph), 7.80 (d, *J* = 11.0 Hz, 2H, Ph), 8.06 (d, *J* = 11.0 Hz, 1H, Ph), 8.10 (d, *J* = 11.0 Hz, 1H, Ph). ^13^C NMR (CDCl_3_): 19.86, 21.41, 45.33, 55.47, 108.80, 113.39, 113.72, 114.99, 124.32, 125.88, 126.99, 127.35, 131.84, 132.25, 132.85, 151.54, 163.56, 168.32. Anal. calcd. for C_19_H_20_N_2_O_3_: C, 70.35; H, 6.21; N, 8.64. Found: C, 70.36; H, 6.28; N, 8.59.

*3-(4-bromo-3-methylbenzoyl)-1-isopropyl-5-methyl-1H-benzo[d]imidazol-2(3H)-one* (**5-25**): white crystalline solid; yield, 67%, mp, 125–127 °C. ^1^H NMR (CDCl_3_): 1.52 (d, *J* = 8.5 Hz, 6H, 2CH_3_), 2.41 (s, 3H, CH_3_), 2.46 (s, 3H, CH_3_), 4.60 (m, 1H, CH), 7.03 (s, 2H, Ph), 7.41 (m, 1H, Ph), 7.77 (m, 2H, Ph), 7.96 (s, 1H, Ph). ^13^C NMR (CDCl_3_): 19.80, 20.26, 22.90, 45.38, 108.88, 115.37, 124.94, 125.28, 125.76, 126.50, 126.79, 127.96, 131.20, 132.03, 132.21, 132.87, 151.80, 170.52. Anal. calcd. for C_19_H_19_BrN_2_O_2_: C, 58.93; H, 4.95; N, 7.23. Found: C, 58.96; H, 4.97; N, 7.20.

*1-isopropyl-3-(4-chloro-3-methylbenzoyl)-5-methyl-1H-benzo[d]imidazol-2(3H)-one* (**5-26**): white crystalline solid; yield, 57%, mp, 163–165 °C. ^1^H NMR (CDCl_3_): 1.54 (d, *J* = 8.5 Hz, 6H, 2CH_3_), 2.29 (s, 3H, CH_3_), 2.62 (s, 3H, CH_3_), 4.70 (m, 1H, CH), 7.03 (s, 2H, Ph), 7.41 (m, 1H, Ph), 7.67~7.73 (m, 2H, Ph), 8.08 (s, 1H, Ph). ^13^C NMR (CDCl_3_): 20.03, 20.12, 22.75, 45.38, 108.80, 115.00, 124.75, 126.40, 126.84, 127.08, 128.84, 129.12, 129.33, 129.76, 132.52, 132.84, 151.50, 171.02. Anal. calcd. for C_19_H_19_ClN_2_O_2_: C, 66.57; H, 5.59; N, 8.17. Found: C, 66.56; H, 5.57; N, 8.20.

*3-(3,5-dimethylbenzoyl)-1-isopropyl-5-methyl-1H-benzo[d]imidazol-2(3H)-one* (**5-27**): white solid; yield, 42%, mp, 120–121 °C. ^1^H NMR (CDCl_3_): 1.54 (d, *J* = 8.5 Hz, 6H, 2CH_3_), 2.17 (s, 3H, CH_3_), 2.23 (s, 3H, CH_3_), 2.55 (s, 3H, CH_3_), 4.60 (m, 1H, CH), 7.09 (s, 2H, Ph), 7.46~7.54 (m, 3H, Ph), 7.96 (s, 1H, Ph). ^13^C NMR (CDCl_3_): 19.75, 21.17, 45.65, 108.97, 115.83, 124.21, 125.28, 125.56, 126.34, 126.68, 127.89, 128.29, 135.41, 136.23, 138.15, 138.60, 151.53, 167.51. Anal. calcd. for C_20_H_22_N_2_O_2_: C, 74.51; H, 6.88; N, 8.69. Found: C, 74.55; H, 6.80; N, 8.70.

*1-isopropyl-5-methyl-3-(thiophene-2-carbonyl)-1H-benzo[d]imidazol-2(3H)-one* (**5-28**): white solid; yield, 53%, mp, 90–92 °C. ^1^H NMR (CDCl_3_): 1.52 (d, *J* = 8.5 Hz, 6H, 2CH_3_), 2.38 (s, 3H, CH_3_), 4.67 (m, 1H, CH), 7.03 (s, 2H, Ph), 7.25 (m, 1H, Thi), 7.73 (m, 2H, Thi), 7.98 (s, 1H, Ph). ^13^C NMR (CDCl_3_): 19.84, 21.39, 45.52, 108.88, 114.98, 122.03, 124.48, 126.45, 126.82, 127.59, 128.48, 134.69, 136.50, 151.27, 167.23. Anal. calcd. for C_16_H_16_N_2_O_2_S: C, 63.98; H, 5.37; N, 9.33. Found: C, 63.88; H, 5.32; N, 9.29.

*1-isopropyl-5-methyl-3-(methylsulfonyl)-1H-benzo[d]imidazol-2(3H)-one* (**5-29**): white solid; yield, 77%, mp, 107–108 °C. ^1^H NMR (CDCl_3_): 1.53 (d, *J* = 8.5 Hz, 6H, 2CH_3_), 2.39 (s, 3H, CH_3_), 3.45 (s, 3H, CH_3_), 4.67 (m, 1H, CH), 7.00 (s, 2H, Ph), 7.65 (s, 1H, Ph). ^13^C NMR (CDCl_3_): 19.82, 21.43, 41.59, 45.70, 109.21, 113.64, 124.49, 126.10, 126.16, 132.25, 150.60. Anal. calcd. for C_12_H_16_N_2_O_3_S: C, 53.71; H, 6.01; N, 10.44. Found: C, 53.69; H, 6.10; N, 10.49.

*3-(4-fluorophenylsulfonyl)-1-isopropyl-5-methyl-1H-benzo[d]imidazol-2(3H)-one* (**5-30**): white solid; yield, 47%, mp, 94–96 °C. ^1^H NMR (CDCl_3_): 1.44 (d, *J* = 8.5 Hz, 6H, 2CH_3_), 2.43 (s, 3H, CH_3_), 4.55 (m, 1H, CH), 6.99 (s, 2H, Ph), 7.20 (t, *J* = 11.0 Hz, 2H, Ph), 7.79 (s, 1H, Ph), 8.17 (m, 2H, Ph). ^13^C NMR (CDCl_3_): 19.74, 21.45, 45.52, 109.20, 116.36, 118.26 (d, *J* = 22.1 Hz), 124.33, 126.70, 127.55, 132.50 (d, *J* = 9.3 Hz), 133.34 (d, *J* = 3.5 Hz), 135.33, 151.60, 165.00 (d, *J* = 255.3 Hz). Anal. calcd. for C_17_H_17_FN_2_O_3_S: C, 58.61; H, 4.92; N, 8.04. Found: C, 58.69; H, 5.00; N, 8.09.

### 3.3. Paper Disc-Diffusion Method

Antibacterial activities of compounds **5-01**~**5-30** were carried out by the method of paper disc-diffusion [20]. The standard bacterial strains, *Bacillus cereus* (1.1846), *Bacillus subtilis* (1.88), *Staphylococcus aureus* (1.89), *Escherichia coli* (1.1574) and *Pseudomonas aeruginosa* (1.2031), were obtained from the China General Microbiological Culture Collection Center. Ampicillin sodium (Sigma, Shanghai, China) was used as positive control. Standardized inoculum (5 × 10^5^ CFU/mL) of each test bacterium was spread on to sterile Müller-Hinton agar (Hangzhou Microbial Reagent Co. Ltd., Zhejiang, China) plates so as to achieve a confluent growth. The title compounds were dissolved in dichloromethane at the concentration of 10 mg/mL, and then, 5 μL of the solutions were transferred onto discs (diameter, 4mm) punched from Whatman no. 1 filter paper, so that the disc contained 50 μg of the compound. After the solvent was evaporated, the sample discs were placed gently on the previously-marked zones in the agar plates. Standard ampicillin (50 μg/disc) paper disc was used as positive controls. The plates were allowed to stand for 1 h at 4 °C for favoring the diffusion and then incubated at 37 °C for 24 h. The diameter of inhibition zone around each disc was measured and recorded at the end of the incubation period. Experiments were repeated in triplicate, and the average values are reported here.

### 3.4. Minimum Inhibitory Concentrations (MICs)

MICs of the title compounds against five strains of bacteria were evaluated by the micro-broth dilution method in 96-well plates [21]. The inoculum was prepared by suspending several colonies from an overnight culture of tested bacteria from 0.5% sheep blood agar media in Müller-Hinton broth and adjusting to a 0.5 McFarland standard (approximately 1.5 × 10^8^ colony-forming units per mL). A further dilution of 1:200 was made by placing 0.25 mL of the adjusted suspension into 49.75 mL of Müller-Hinton broth. The tested compounds were firstly dissolved in dimethyl sulfoxide (DMSO) at the concentration of 10 mg/mL, and it was diluted ten-fold with sterile water to give the stock solution. Two-fold serial dilutions of the tested compounds were prepared in Müller-Hinton broth. Then, the dilutions and inoculated suspension of the bacteria were delivered to wells of a 96-well plate at the ratio of 1:1. The final concentration of inoculum in each well was 3.7 × 10^5^ colony-forming units per mL. After incubation for 24 h at 30 °C, the MICs were examined. Experiments were repeated in triplicate, and standard ampicillin was used as the positive control.

### 3.5. Spore Germination Assay

The tested compounds (10 mg) dissolved in dimethyl sulfoxide (DMSO, 1 mL) were diluted 100-times with sterile distilled water to afford tested solution (100 μg/mL). The samples were inoculated with aliquots of spore suspension of *B. cinerea* containing 1.0 × 10^6^ spores/mL. Aliquots of 10 μL of prepared spore suspension were placed on separate glass slides in triplicate. Slides containing the spores were incubated in a moisture chamber at 25 °C for 6 h. Each slide was then observed under the microscope for spore germination. The spore-generated germ tubes were enumerated, and percentage of spore germination was calculated. Azoxystrobin was preferred as the positive control [22].

To determine the median effective concentration (EC_50_, μg/mL) of the compounds, which exhibited strong antifungal activity, the tested samples were further diluted with distilled water to the desired concentration range, and the final solution was separately tested for spore germination of *B. cinerea*, as described above. The experimental data of the fungicidal activities were analyzed using SPSS 13.0 for Windows.

## 4. Conclusions

In summary, a series of new 3-acyl-1-isopropyl-5-methyl-benzimidazolone derivatives have been synthesized and evaluated as potential antimicrobial agents *in vitro*. The bioassay of these analogues showed that sulfonic acids and most of carboxylic acids derivatives were not effective against tested strains. Among these compounds, analogues **5-07** and **5-19** were the most interesting candidates as antibacterial agents; **5-02**, **5-07**, **5-12**, **5-15**, **5-19**, **5-20** and **5-25** were considered lead compounds worthy of further structural optimization and development as agricultural fungicide. The SAR revealed that the steric effect may play a vital role for the antimicrobial activity of aliphatic acid derivatives, and the type and position of substituted groups at benzoic acid have notable influence on the activity of aromatic acid derivatives. Additionally, comparing 1-isopropenyl-benzimidazol-2-one that we reported previously, the introduction of a methyl group could affected the activity obviously. On the basis of the SAR study, the synthesis of analogues of lead compounds **5-07** and **5-19**, in order to search for more potent activity, is currently in progress.

## Figures and Tables

**Table 1:** Antimicrobial activity of title compounds.

Compounds	Zone of inhibition (mm)^a^					Inhibition rate (%)^b^
	
	*B. cereus*	*B. subtilis*	*S. aureus*	*E. coli*	*P. aeruginosa*	*B. cinerea*
**5-01**	—	—	—	—	—	40.38
**5-02**	11 (+++)	10 (+++)	10 (+)	10 (++)	11 (+)	71.54
**5-03**	—	—	—	—	—	37.06
**5-04**	—	—	—	—	—	51.92
**5-05**	—	—	—	—	—	43.24
**5-06**	—	—	—	—	—	47.69
**5-07**	14 (+++)	16 (+++)	12 (+++)	11 (++)	10 (++)	80.58
**5-08**	—	—	—	—	—	38.46
**5-09**	—	—	—	—	—	47.12
**5-10**	—	—	—	—	—	29.71
**5-11**	—	—	—	—	—	41.28
**5-12**	10 (++)	8 (++)	—	—	—	93.65
**5-13**	—	—	—	—	—	17.65
**5-14**	13 (++)	11 (++)	8 (++)	—	—	44.12
**5-15**	15 (+++)	15 (+++)	12 (+++)	10 (+)	10 (++)	73.18
**5-16**	—	—	—	—	—	17.06
**5-17**	10 (++)	12 (++)	11 (++)	—	—	47.31
**5-18**	—	—	—	—	—	53.08
**5-19**	17 (+++)	15 (+++)	10 (+++)	11 (++)	11 (++)	88.46
**5-20**	9 (++)	8 (+++)	8 (++)	11 (+)	9 (++)	75.77
**5-21**	8 (++)	9 (++)	9 (++)	—	—	44.87
**5-22**	8 (++)	10 (++)	11 (++)	—	—	48.08
**5-23**	—	—	—	—	—	45.88
**5-24**	—	—	—	—	—	43.24
**5-25**	9 (++)	9 (++)	11 (++)	11 (+)	11 (+)	77.95
**5-26**	11 (++)	10 (++)	12 (+)	11 (+)	10 (+)	32.35
**5-27**	—	—	—	—	—	55.00
**5-28**	—	—	—	—	—	42.35
**5-29**	—	—	—	—	—	42.31
**5-30**	—	—	—	—	—	47.44
Ampicillin	25 (+++)	26 (+++)	20 (+++)	18 (++)	25 (+++)	/
Azoxystrobin	/	/	/	/	/	100.00

All values are the means of three replicates.

a “+”: eyeable; “++”: clear; “+++”: transparent; “—“: invisible; “/”: not tested; (Dose: 50 μg/disc);

b Inhibition rate were tested at the concentration of 25 μg/mL.

**Table 2:** Minimum inhibitory concentrations (MICs) and 50% effect concentrations (EC_50_) of title compounds against standard strains.

Compounds	MICs (μg/mL)					EC_50_ (μg/mL)
	
	*B. cereus*	*B. subtilis*	*S. aureus*	*E. coli*	*P. aeruginosa*	*B. cinerea*
**5-02**	100	100	100	>100	>100	21.07
**5-07**	25.0	12.5	25.0	50.0	100.0	17.62
**5-12**	100	100	100	>100	>100	10.68
**5-14**	100	100	100	>100	>100	/
**5-15**	50.0	50.0	100.0	100.0	100.0	19.75
**5-17**	100	>100	>100	>100	>100	/
**5-19**	6.25	12.5	12.5	50.0	100.0	14.23
**5-20**	100	>100	>100	>100	>100	17.33
**5-21**	100	>100	>100	>100	>100	/
**5-22**	100	>100	>100	>100	>100	/
**5-25**	100	>100	>100	>100	>100	16.39
**5-26**	100	>100	>100	>100	>100	/
Ampicillin	12.5	3.13	6.25	3.13	25.0	/
Azoxystrobin	/	/	/	/	/	1.53
